# Hybrid Magneto-Plasmonic Nanostructures for Enhanced Dual-Mode Hyperthermia

**DOI:** 10.3390/nano16150938

**Published:** 2026-07-29

**Authors:** Amirhossein Sanchooli, Patricia de la Presa

**Affiliations:** 1 Department of Physics, Faculty of Science, University of Kurdistan, Sanandaj 66177-15175, Iran; amirhossiensanchooli@gmail.com; 2 Institute of Applied Magnetism, Complutense University of Madrid, A6 22.500 km, 28230 Las Rozas, Spain; 3 Department of Material Physics, Complutense University of Madrid, 28040 Madrid, Spain

**Keywords:** magnetic nanoparticles, gold nanorods, magneto-plasmonic hybrid nanostructures, dual-mode hyperthermia, photothermal and magnetic heating

## Abstract

This study addresses a major challenge in hyperthermia therapy: achieving fast and efficient heat generation in target tissues. A novel magneto-plasmonic nanostructure is developed by combining gold nanorods (GNRs) and iron oxide nanoparticles (IONPs), each synthesized independently to compare their individual and combined heating performance. A key innovation was the use of (3-mercaptopropyl)trimethoxysilane (MPTMS) as a covalent linker, enabling the stable integration of both components into a single hybrid system. Under simultaneous near-infrared (NIR) laser and alternating magnetic field exposure, the hybrid nanostructure exhibited a rapid and intense temperature rise, surpassing the effects of either material alone. A control sample consisting of a physical mixture of the two components (GNR + IONP) reached SAR values comparable to those of the covalently linked hybrid (GNR + MPTMS + IONP) under simultaneous excitation, the two being equal within experimental error. Although the chemical linkage modifies the optical absorbance of the GNRs and may partially restrict the Brownian relaxation of the magnetic nanoparticles, these effects do not translate into a measurable loss of heating efficiency under combined activation. Importantly, the covalent linkage yields a robust, structurally stable assembly whose components move together—an essential requirement for functionalities such as magnetic guidance and targeted delivery of the whole nanostructure, which a simple physical mixture cannot provide. As a physicochemical proof of concept, these results establish the dual-mode heating performance of the hybrid nanostructure in aqueous suspension and motivate the biological evaluation required for any future therapeutic use.

## 1. Introduction

Hyperthermia is a cancer treatment method based on increasing the temperature of living tissue to destroy tumors [1]. By localizing the treatment of hyperthermia, damage to the healthy tissues of the living organism can be prevented [2]. Nanotechnology enables more efficient and precise localized hyperthermia, minimizing damage to healthy cells [3]. Developments in the synthesis of various nanoparticles have led to the production of nanoparticles that can release heat when interacting with an alternating magnetic field (AMF), releasing this heat to their immediate surroundings [4,5].

The most common nano heaters in local cancer treatment are magnetic and plasmonic nanoparticles [6,7]. Magnetic nanoparticles release heat when interacting with an AMF due to hysteresis loss, and this method is used to raise the temperature in biomedical applications for the local treatment of cancer cells, which is called Magnetic Hyperthermia (MHT) [5,6]. Another method involves using metallic nanoparticles to produce heat when interacting with IR light due to plasmonic effects; this strategy, also applied to the local treatment of cancer cells, is called Photothermal Therapy (PTT) [4,8]. In MHT, achieving a high Specific Absorption Rate (SAR) is crucial for effective treatment. The SAR of magnetic nanoparticles is primarily influenced by several factors, including their magnetic properties (such as saturation magnetization and magnetic anisotropy constant), particle size, the intensity and frequency of the applied magnetic field, and the nanoparticle concentration [9,10]. An important issue limiting the application of the MHT is the amplitude and frequency of the magnetic field required to produce the heating, which should be within the range approved for human use to avoid overcoming the discomfort factor limit H_0_ × f < 5 × 10^9^ A·m^−1^·s^−1^, called the Atkinson–Brezovich limit [11]. Moreover, this limit has been decreased by a factor of 3 by Pilpilidis et al. [12]. Another practical obstacle of MHT in clinical terms is that the SAR decreases significantly when the magnetic nanoparticles are absorbed by cancer cells [13]. These limitations in magnetic hyperthermia show that there is a need to produce nanoparticles that have high SAR efficiency at reduced fields.

On the other hand, in recent years, PTT has emerged as a promising technique for local cancer treatment [8,14,15]. In this process, light-sensitive agents convert photon energy into heat due to the plasmonic effects of metal nanoparticles. Metal nanoparticles generate heat upon interaction with infrared laser radiation through a phenomenon known as localized surface plasmon resonance (LSPR). This occurs when the electric field of the laser light induces coherent oscillations of the metal’s free electrons. These oscillations subsequently transfer energy to the atomic lattice in the form of phonons, resulting in heat generation that is then transferred to the surrounding medium [16]. Various particles, such as gold, platinum, silver, and copper nanoparticles, have been used for the photothermal process [17,18,19,20]. Among the various nanoparticles employed in photothermal therapy, gold nanoparticles have attracted significant attention due to their unique and advantageous properties. These include the following: (i) A suitable surface chemistry that allows for easy functionalization with thiol and amine groups, enabling the attachment of targeting antibodies or therapeutic agents. (ii) Excellent biocompatibility, making them safe for biomedical applications. (iii) A well-defined and tunable LSPR, which enhances their efficiency in converting light into heat under near-infrared irradiation [21]. However, although photothermal therapy (PTT) typically operates within the biological optical windows—mainly the first (NIR-I, 650–950 nm) and second (NIR-II, 1000–1350 nm), where light absorption by tissues is minimized and therapeutic irradiation doses remain within biologically safe ranges—the low penetration depth of NIR light (approximately 3–5 mm in most soft tissues) is still insufficient for the treatment of deep-seated tumors. This limited penetration, primarily caused by strong light scattering and residual absorption in biological media, remains one of the major constraints of PTT for biomedical applications [22].

As previously discussed, both magnetic and photothermal hyperthermia approaches face limitations in achieving high heating efficiency, either due to the discomfort threshold associated with magnetic hyperthermia or the limited tissue penetration depth of photothermal therapy [13,22,23]. In order to overcome these limitations, the combination of MHT and PTT methods can be used. With this approach, by applying a magnetic field and laser radiation at the same time, high temperatures can be reached in a short time to destroy cancer cells. Simultaneous magnetic and photothermal hyperthermia requires nanoparticles that respond to both a magnetic field and infrared radiation; magneto-plasmonic hybrid nanoparticles are therefore a promising solution for enhancing SAR. Building on this concept, magneto-plasmonic hybrids that couple a plasmonic gold or silver component with magnetic nanoparticles have been specifically explored for dual-mode hyperthermia, in which magnetic and photothermal heating are combined to increase the overall efficiency [13,24,25,26,27,28]. Espinosa et al. [13,26] exploited the duality of iron oxide nanoparticles (IONPs) to combine magnetic hyperthermia and photothermal heating, showing that bimodal treatment amplifies heating efficiency relative to either mechanism alone. In the same line, Das et al. [24] reported Ag/Fe_3_O_4_ nanoflowers that reach markedly higher heating rates when an alternating magnetic field and near-infrared irradiation are applied simultaneously, while Ovejero et al. [28] synthetized IONPs linked to gold nanorods (GNRs) by a silica shell capable of combined magnetic and plasmonic hyperthermia. More recently, Lázaro et al. [27] combined magnetic hyperthermia and photothermia using polyelectrolyte/gold-coated magnetic nanorods, and El-Boubbou et al. [25] developed PEGylated opto-magnetic gold–iron oxide nanoprobes for synergistic photothermal therapy. Collectively, these works establish combined magnetic–photothermal hyperthermia as an effective and increasingly mature strategy. In most of them, however, the plasmonic and magnetic functionalities are brought together as physical mixtures, core–shell coatings, or co-assembled structures. A covalent linkage that binds discrete GNRs and IONPs into a single, magnetically manipulable entity—so that both components move together and can be simultaneously activated by light and by an alternating magnetic field—remains comparatively unexplored and constitutes the focus of the present work.

Spherical gold and silver nanoparticles used as cores exhibit a plasmonic absorption peak in the visible to near-infrared region. Specifically, spherical gold nanoparticles typically show an absorption peak between 500 and 525 nm, depending on their size. This optical property makes them particularly useful for photothermal applications, as they efficiently absorb light and convert it into heat, enabling effective temperature elevation in targeted therapies [15,21,29,30]. However, these wavelengths are not recommended for biological applications [31]. Nevertheless, when nanoparticles are in the form of GNRs with a length of less than 100 nanometers, their absorption peaks shift into NIR, which is the appropriate wavelength region for biological applications [32,33,34]. Through the use of anisotropic plasmonic GNRs as cores, this research achieves improved optical absorption in magneto-plasmonic hybrid nanostructures, representing a significant advancement in the field. The attachment of IONPs to GNRs yields a hybrid nanostructure, where the anisotropic rod shape of the gold provides superior infrared absorption. This hybrid system enables dual activation by alternating magnetic fields and infrared laser light, leading to efficient heat production.

Various methods exist for hybridizing GNRs with metal oxides [30]. In this work, (3-mercaptopropyl)trimethoxysilane (MPTMS) was employed as a bifunctional linker. Its thiol group (–SH) has a strong affinity for gold, forming a stable covalent bond to the GNR surface [35,36], while its methoxy groups (–OCH_3_) hydrolyze into silanol groups (–Si–OH) that anchor the IONPs [37]. MPTMS thus bridges the two components, connecting the GNRs to the IONPs.

Our research centers on developing a unique magneto-plasmonic hybrid nanostructure that successfully conjugates GNRs to IONPs. This stable and targeted conjugation was successfully established using the MPTMS chemical linker. The system’s most critical feature is its dual functionality: when the nanostructure is simultaneously activated by near-infrared light and an alternating magnetic field, its heating efficiency is markedly enhanced within a short time. This rapid and pronounced temperature increase is a desirable feature for time-critical hyperthermia and identifies this hybrid as a physicochemically validated platform whose biological performance remains to be assessed.

## 2. Experimental

### 2.1. Gold Nanorod Synthesis

The GNRs were synthesized via a seed-mediated growth method in two steps. First, gold seeds were prepared by dissolving 364.4 mg CTAB in 5 mL of water and adding 5 mL of 0.5 mM HAuCl_4_. Seed growth was initiated by the rapid addition of 600 μL of 10 mM NaBH 4 (at 4 °C), followed by stirring for 20 min. Next, the growth solution was prepared by dissolving 182.2 mg CTAB and 22 mg hydroquinone in 5 mL of water and combining it with 200 μL of 4 mM AgNO_3_ and 5 mL of 1 mM HAuCl_4_. The reaction was started by adding 12 μL of the seed suspension and monitored via UV–Vis spectroscopy until the spectrum stabilized, followed by purification via centrifugation at 10,000× *g* for 10 min [38,39,40].

### 2.2. Iron Oxide Nanoparticle Synthesis

Magnetite (Fe_3_O_4_) nanoparticles were synthesized by coprecipitation of a mixture of FeCl_3_·6H_2_O (0.09 mol) and FeCl_2_·4H_2_O (0.054 mol) in 425 mL of aqueous solution with 75 mL of an alkaline medium. The particle size was controlled by adjusting the base type, addition rate, and aging time. To obtain γ-Fe_2_O_3_, HNO_3_ (2 M) was added to the particles, and the mixture was stirred for 15 min. The nitric acid was then removed by magnetic decantation, and 75 mL of Fe(NO_3_)_3_ (1 M) and 130 mL of water were added to the particles. The mixture was heated to boiling and stirred for 30 min. The particles were then cooled to room temperature and, by magnetic decantation, the supernatant was replaced with 300 mL of HNO_3_ (2 M), and the suspension was stirred for 15 min. Finally, the particles were washed several times with acetone and redispersed in water. A rotary evaporator was used to remove residual acetone and concentrate the sample [41,42,43].

### 2.3. Hybrid Nanoparticle Synthesis

GNRs (1.5 mg·mL^−1^) were purified by two consecutive centrifugation cycles (10,000 rpm, 10 min) to remove excess cetyltrimethylammonium bromide (CTAB) and redispersed in deionized (DI) water.

For functionalization, 10 mL of the GNR suspension was mixed with 6 mL of ethanol, and 10 μL of MPTMS was added. The mixture was stirred at room temperature for 2 h, allowing the thiol groups to anchor onto the gold surface and the silane groups to undergo partial hydrolysis. Subsequently, 90 μL of aqueous ammonia (25%) was added dropwise to adjust the pH to ~8, and stirring was continued for a further 2 h at room temperature to promote the complete hydrolysis of the methoxysilane groups into silanols. The MPTMS-functionalized GNRs were collected by centrifugation (10,000 rpm, 10 min), washed once to eliminate unbound MPTMS, and redispersed in 2 mL of ethanol.

Then, 10 mL of a γ-Fe_2_O_3_ suspension (1.5 mg·mL^−1^ in DI water) was added dropwise to the MPTMS-functionalized GNR dispersion under continuous stirring. The mixture was kept under stirring at 50 °C for 3 h to facilitate the formation of Fe–O–Si linkages. The resulting hybrid nanostructures were collected by centrifugation (7000 rpm, 15 min), washed five times with DI water and ethanol, and finally redispersed in DI water for characterization.

For comparative purposes, a control sample was prepared consisting of a physical mixture of GNRs and γ-Fe_2_O_3_ nanoparticles at the same concentrations, without any chemical linker.

## 3. Experimental Methods

UV–Vis Spectroscopy: UV–Vis spectra were recorded using a UV-1100 spectrophotometer (NADE) equipped with a deuterium and tungsten lamp. Samples were prepared in deionized water and measured in the wavelength range of 200–1100 nm with a spectral bandwidth of 4 nm.

X-ray Diffraction (XRD): Powder XRD patterns were collected using a Bruker D8 Advance diffractometer with a copper X-ray source (Kα, λ = 1.5406 Å). Data were acquired in the 2θ range of 10–120° with a step size of 0.019° and a collection time of 298.40 s per step.

Phase Identification: Phase identification was performed using a Bruker D8 Discover diffractometer equipped with a Co Kα source (λ = 1.78897 Å) and a LynxEye linear detector. Data were collected in the 2θ range of 10–120° with a step size of 0.015°. Rietveld refinement was performed using TOPAS v6.0 (Bruker AXS) to analyze the XRD data.

Transmission Electron Microscopy (TEM): TEM measurements were carried out using a JEM-1400 Plus high-contrast TEM operating at an acceleration voltage of 40–120 kV at the Spanish National Center for Electron Microscopy (ICTS), Complutense University of Madrid, Spain. Particle dimensions were measured manually from calibrated TEM micrographs using ImageJ (version 1.4.3.67), and the resulting size distributions were fitted to a lognormal function. In the case of the hybrid, the suspension imaged was that obtained after the complete purification procedure described above (five consecutive washing/centrifugation cycles with DI water and ethanol), so that all TEM images of the hybrid correspond to the washed material.

Magnetometry: Magnetic properties were characterized using a SQUID magnetometer equipped with an Ever-Cool system for continuous operation without helium transfer. The system provides a temperature range of 1.9–400 K and a maximum magnetic field of ±4000 kA/m. Hysteresis cycles have been measured at 5 and 300 K, and zero-field-cooled (ZFC) and field-cooled (FC) curves have been measured from 5 to 300 K, with an applied field of 7.9 kA/m.

MHT: Two instruments were used for this characterization. The first was an Ambrell EasyHeat system operating at 220 kHz, with a magnetic-field amplitude of 5.7 to 49.4 kA/m; the second was the commercial Magnetherm 1.5 system (Nanotherics), with frequencies from 111 to 900 kHz and field amplitudes up to 14.9 kA/m.

PTT: Newish Laser Module operating at 808 nm with a power of 200 mW and 0.8 W/cm^2^ laser intensity was used for photothermal characterization. The laser beam was directed perpendicularly onto the sample, which was placed in a Teflon holder. The temperature rise during the heating experiments was monitored using a FLIR Model E53 Thermal Imaging Camera with a frame rate of 30 frames per second [44].

## 4. Results and Discussion

### 4.1. Structural, Optical, and Magnetic Characterization

**Figure 1:**
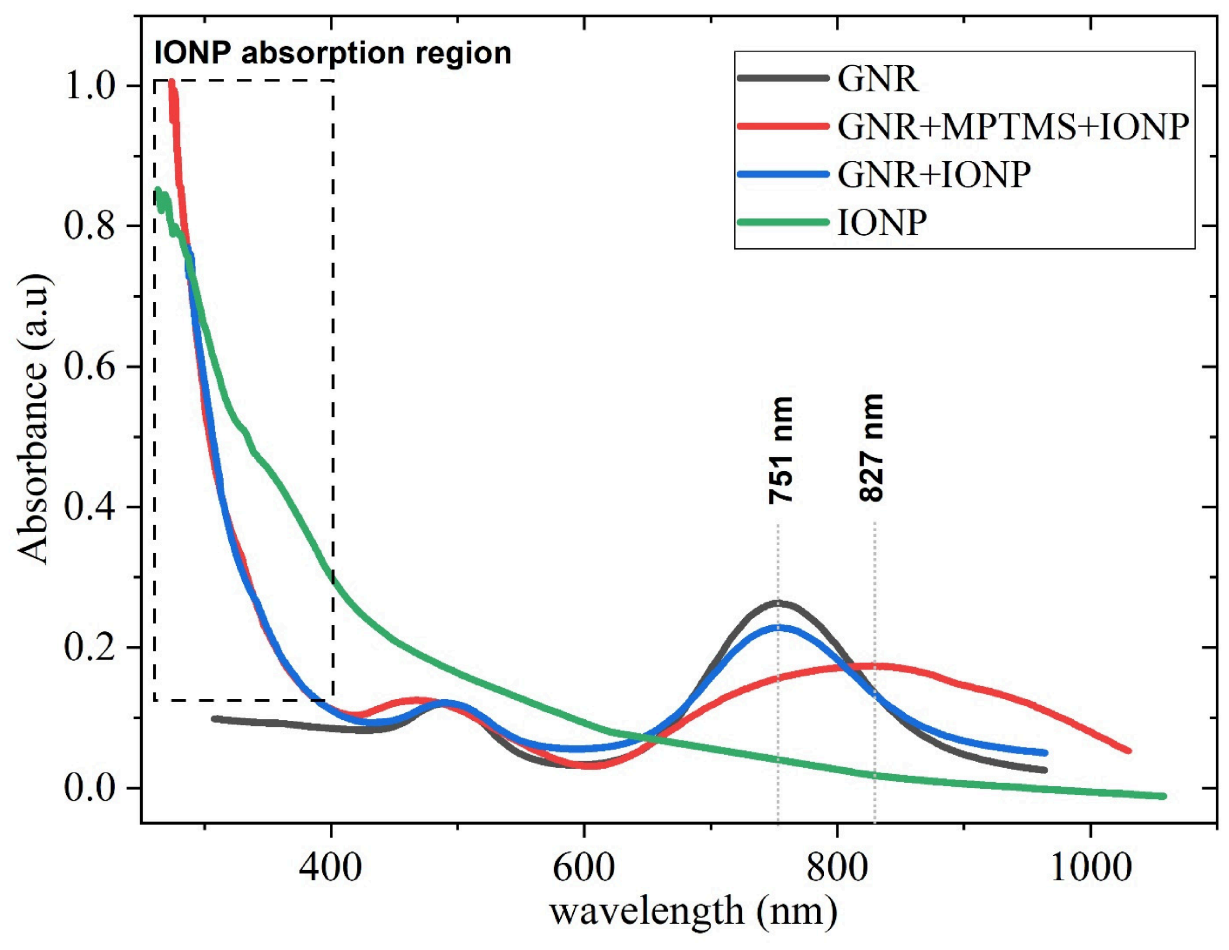
Normalized UV–Vis absorption spectra of: (black) bare GNRs, (red) GNRs conjugated to IONPs via MPTMS linker, (blue) a mixture of GNRs and IONPs, (green) IONPs. The circled part corresponds to the region of highest absorption of IONPs.

To ensure the successful formation of this hybrid nanostructure, the absorption spectra were measured. GNRs exhibit two absorption peaks in their absorption spectra due to transverse and longitudinal plasmonic oscillations, which is a characteristic feature of GNRs [32,34,38,39]. The unique absorption spectrum of GNRs allows for the confirmation of successful synthesis. The absorption spectrum of the synthesized GNRs (Figure 1) exhibited peaks at 495 nm and 751 nm, corresponding to transverse and longitudinal plasmon resonances, respectively. This absorption spectrum is consistent with previously reported data for bare GNRs [40]. As shown in Figure 1, the 495 nm absorption peak corresponding to the transverse plasmon resonance of the bare GNRs shifts to lower wavelengths in the GNR + MPTMS + IONP hybrid. In contrast, the longitudinal plasmon resonance undergoes a redshift from 751 nm to 827 nm. Both results indicate successful conjugation of IONPs to GNRs [45,46,47]. Also, in order to create a better concept of the attached and unattached state of IONPs to GNRs, another sample was prepared by simply combining GNRs with IONPs, without a linker (Figure 1; blue graph). Comparison of the blue and black spectra in Figure 1 revealed that, in the absence of a linker, the GNR + IONP mixture exhibited the absorption band from 275 nm to 420 nm due to IONPs, but no red-shift in either transversal or longitudinal plasmon resonance peak of the GNRs was observed. For the sake of clarity, the absorption spectrum of IONPs was also acquired (Figure 1, green spectrum). As expected, it showed no significant absorption in the 500–1000 nm range.

The IONPs were characterized with X-ray diffraction by means of a Co source for quantitative Rietveld analysis. The lattice parameter of perfect magnetite is 8.396 Å, and for pure maghemite is 8.3515 Å. The cell parameter obtained from the synthesized samples was 8.356 ± 0.002 Å, as determined by Rietveld analysis. In addition, the near absence of divalent cations in both octahedral and tetrahedral sites suggests that the sample is an almost perfectly stoichiometric maghemite (γ-Fe_2_O_3_).

**Figure 2:**
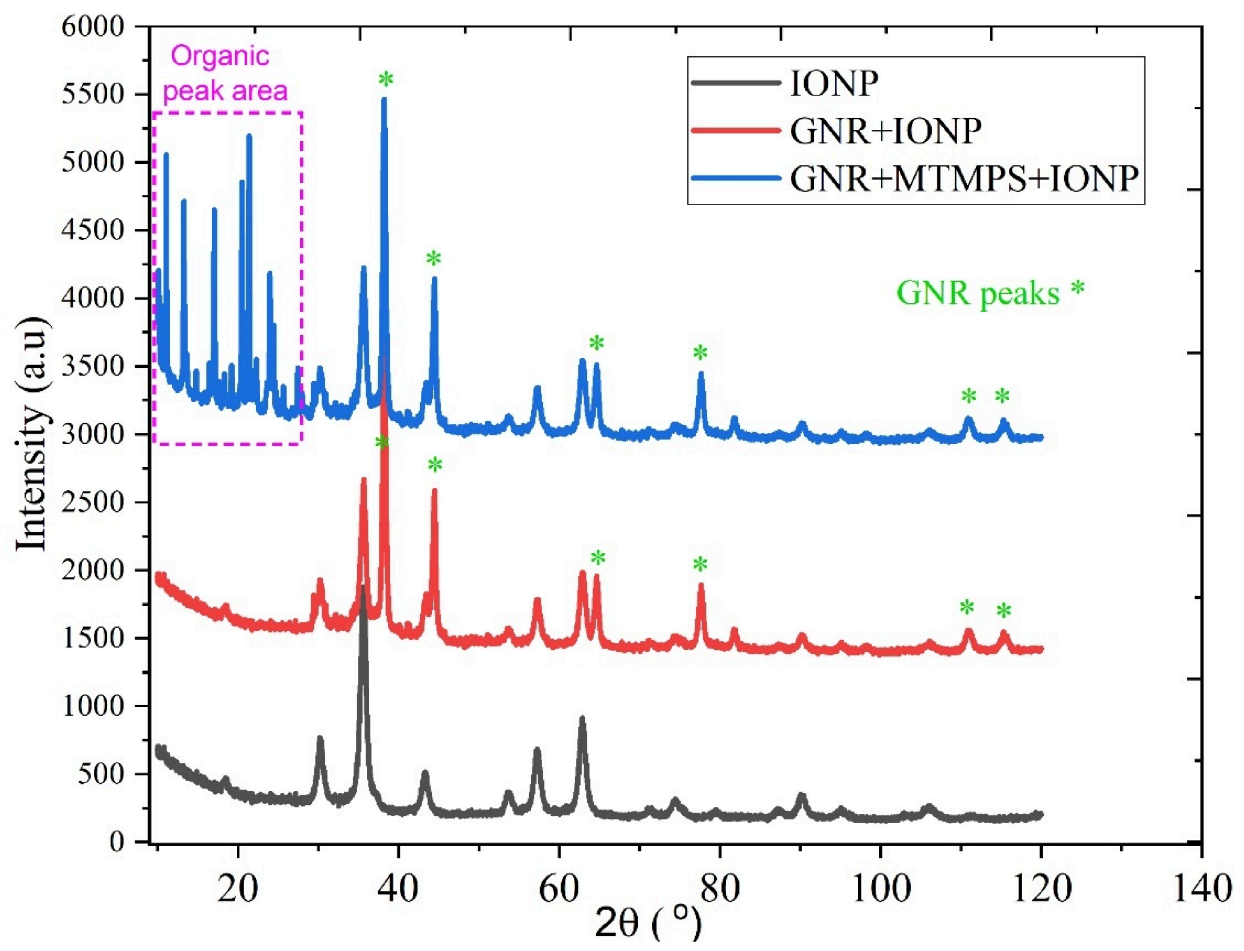
XRD pattern of iron oxide nanoparticles (black graph), iron oxide nanoparticles mixed only (red graph), GNRs with MPTMS ligand and iron oxide nanoparticles (blue graph).

To understand the elemental structure of these hybrid nanoparticles, the X-ray diffraction pattern was measured with a Cu source (Figure 2).

Figure 2 displays the XRD patterns, revealing six characteristic peaks for γ-Fe_2_O_3_ indexed as (220), (311), (400), (422), (511), and (440), corresponding to 2θ values of 30.17°, 35.52°, 43.15°, 53.66°, 57.18°, and 62.94°, respectively [48,49,50]. The presence of sharp peaks in the XRD patterns indicates a high degree of crystallinity of the nanoparticles.

Comparison of these patterns with the IONPs reference spectrum reveals the distinct presence of GNRs in both GNR + IONP and GNR + MPTMS + IONP samples. Characteristic peaks for GNRs are marked with an asterisk (*) and correspond to the (111), (200), (220), and (311) planes at 38.21°, 44.39°, 64.52°, and 77.55°, respectively.

The GNR + MPTMS +IONP sample exhibits additional peaks at angles below 30°, attributed to the presence of organic ligands. This confirms a clear difference between the two samples.

**Figure 3:**
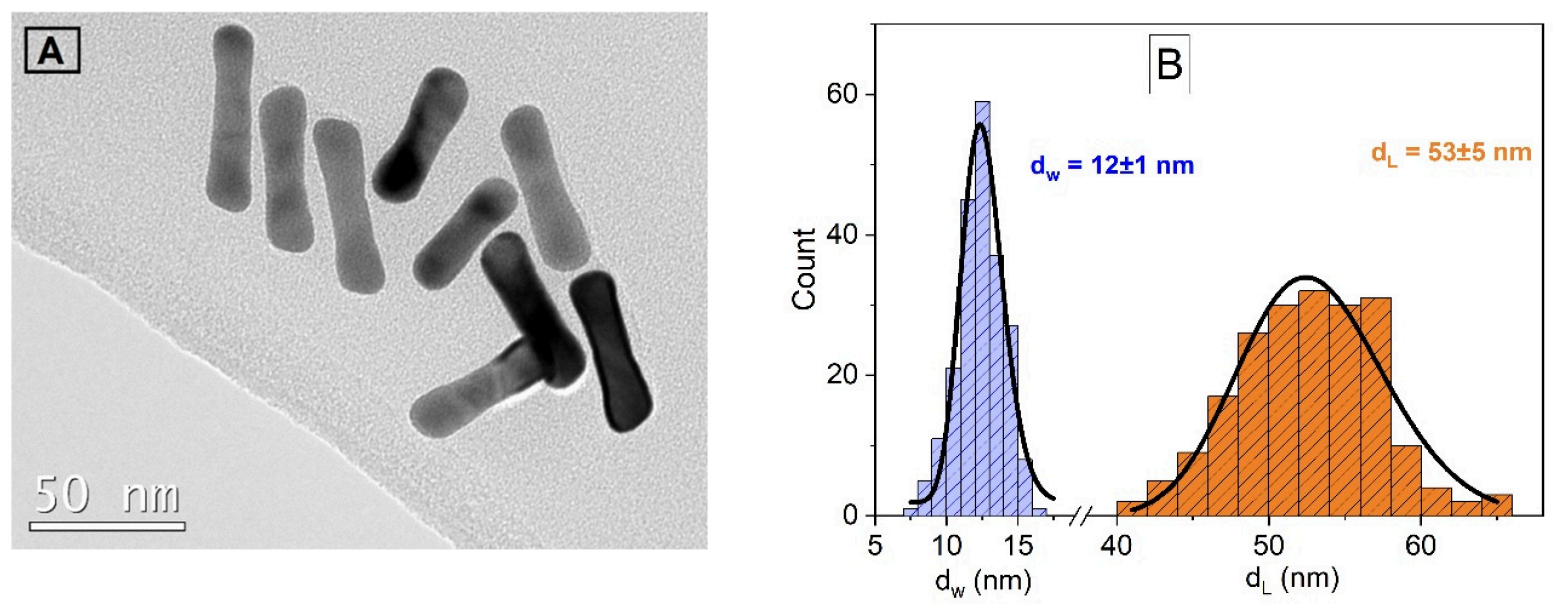
(**A**) Representative TEM image of bare GNRs. (**B**) Particle size distributions of GNR length (orange histogram) and width (blue histogram), determined from measurements of more than 200 particles. Further micrographs are shown in Figure S1 of the Supporting Information.

**Figure 4:**
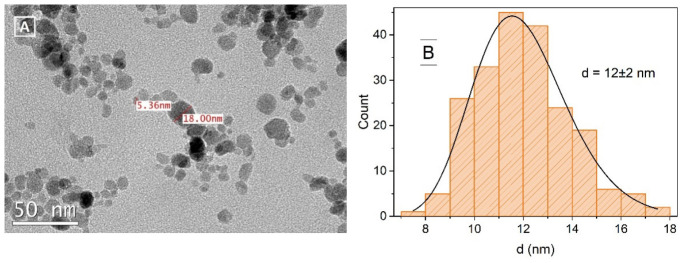
(**A**) TEM image of γ-Fe_2_O_3_ nanoparticles alone. (**B**) Mean particle size distribution taken by the measurement of more than 200 nanoparticles. Further micrographs are shown in Figure S2 of the Supporting Information.

To confirm the successful synthesis of the hybrid nanostructure, the samples were analyzed using Transmission Electron Microscopy (TEM). Figure 3 shows bare GNRs with their particle size distribution, and Figure 4 depicts isolated IONPs. The size distributions of both components were determined from TEM micrographs by fitting the corresponding histograms to a lognormal function. Representative micrographs of both components are shown in the Supporting Information (Figure S1 for the bare GNRs and Figure S2 for the γ-Fe_2_O_3_ nanoparticles). The size distributions were obtained from a larger set of 8 and 6 independent micrographs, respectively, from which more than 200 particles were measured in each case. The GNRs exhibit a median length of L = 53 ± 5 nm (w = 0.09) and a median diameter of d = 12 ± 1 nm (w = 0.10), yielding an average aspect ratio of approximately 4.4, consistent with a longitudinal plasmon resonance located in the first biological window. Both dimensions correspond to relative polydispersities (σ/L and σ/d) of approximately 9–10%, evidencing a highly monodisperse population in both length and diameter. The γ-Fe_2_O_3_ nanoparticles show a median diameter of d = 12 ± 2 nm (w = 0.16), with a relative polydispersity of about 16%. All values fall well below the ~20% threshold commonly associated with narrow size distributions in colloidal nanoparticle systems, confirming the good size homogeneity achieved by the synthesis protocols. The narrow size distribution of the γ-Fe_2_O_3_ nanoparticles is particularly relevant for magnetic hyperthermia performance, since the specific absorption rate is highly sensitive to particle size dispersion, with polydispersity broadening the distribution of relaxation times and reducing the heating efficiency at a given field amplitude and frequency. Likewise, the low-dimensional dispersion of the GNRs in both length and diameter ensures a well-defined longitudinal plasmon band, minimizing inhomogeneous broadening of the NIR absorption and thus favoring efficient photothermal conversion at the 808 nm excitation wavelength.

**Figure 5:**
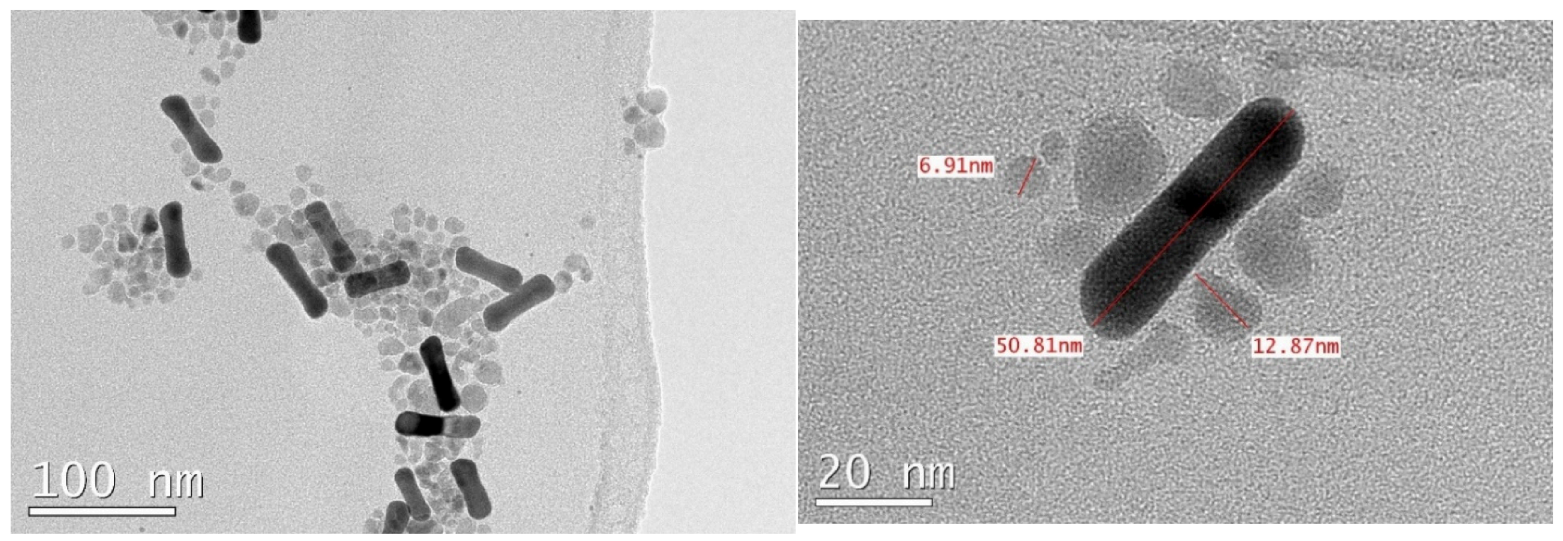
TEM images of GNRs with MPTMS ligand and γ-Fe_2_O_3_ nanoparticles at different scales. The sample was imaged after the complete washing protocol; further micrographs are shown in Figure S3 of the Supporting Information.

**Figure 6:**
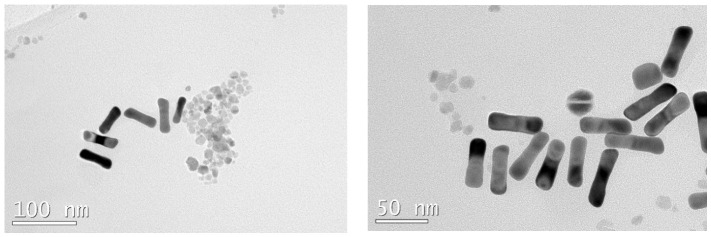
TEM image of GNRs and γ-Fe_2_O_3_ nanoparticles, which were mixed only (no chemical attachment) in different scales. Further micrographs are shown in Figure S4 of the Supporting Information.

Figure 5, obtained at different magnifications, shows γ-Fe_2_O_3_ nanoparticles decorating the GNRs, in an irregular distribution consistent with the non-site-selective MPTMS coupling, thereby evidencing the successful conjugation mediated by the MPTMS linker. In contrast, Figure 6, which presents a simple physical mixture of GNRs and IONPs in the absence of the linker, shows no discernible association between the two components. These observations support, together with the UV–Vis, XRD, and magnetometry data, the formation of the hybrid nanostructure through MPTMS-mediated covalent linkage.

**Figure 7:**
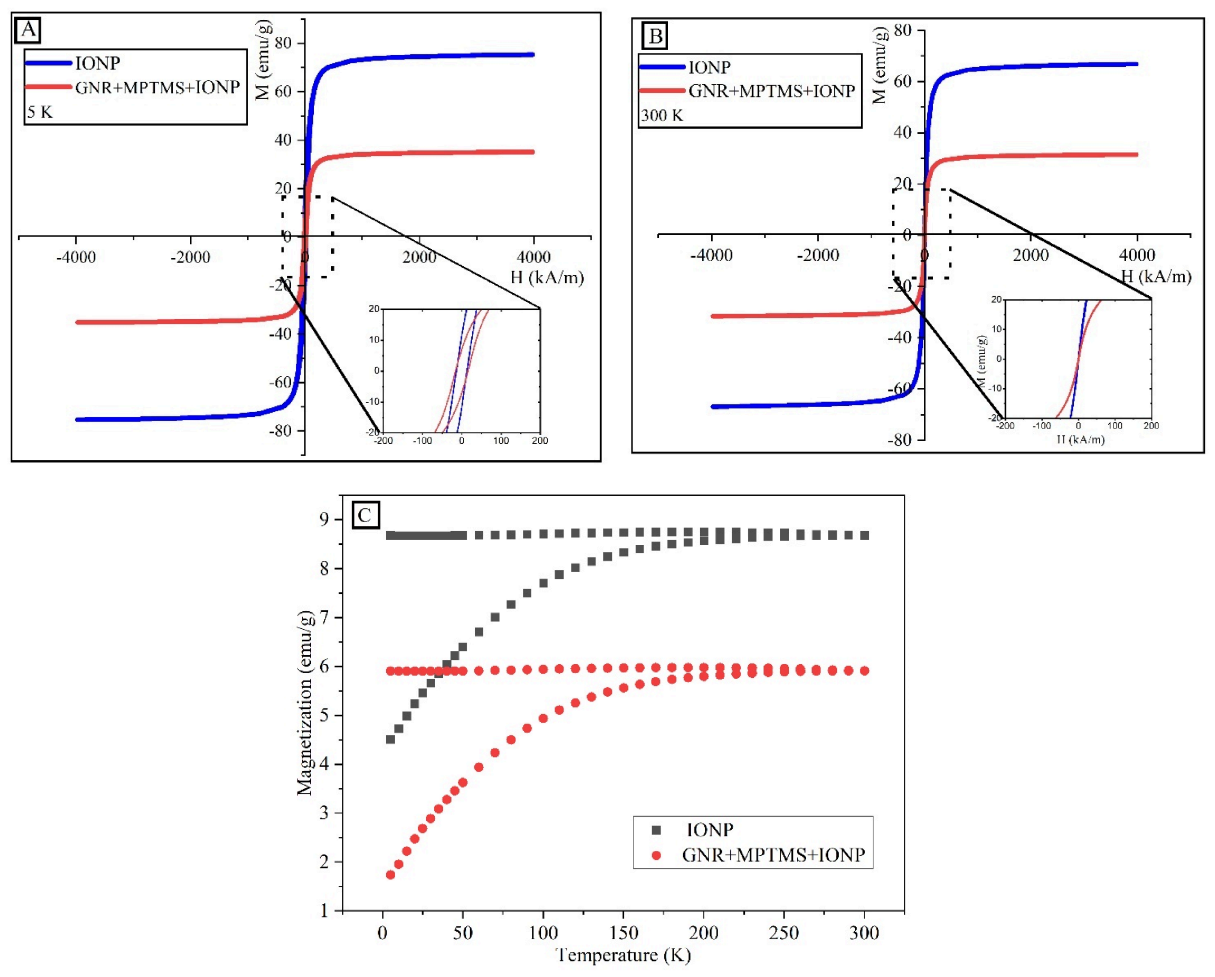
Hysteresis cycles for γ-Fe_2_O_3_ nanoparticles and γ-Fe_2_O_3_ nanoparticles attached to GNRs at 5K (**A**) and 300K (**B**), (**C**) ZFC-FC at 7.9 kA/m applied field.

The magnetic hysteresis loop, presented in Figure 7A, confirms the ferromagnetic behavior of the nanoparticles at 5 K. Within the magnetic field range of −200 kA/m to 200 kA/m, the loop exhibits a coercivity (Hc) of 12 kA/m. This non-zero coercivity clearly indicates the presence of permanent magnetization at low temperature. γ-Fe_2_O_3_ nanoparticles exhibit 75 emu/g saturation magnetization, implying a strong magnetic moment when fully aligned in an external field [51,52]. The saturation magnetization of the hybrid nanostructure is 32 emu/g, smaller than that of the bare IONPs because of the presence of diamagnetic GNRs [53,54]. No change in the coercive field of the hybrid structure is observed, indicating that the interactions between gold and IONPs are not strong enough to cause significant changes in the coercivity. On the other hand, at 300 K, the coercivity approaches zero, consistent with superparamagnetic behavior at room temperature.

Based on the ZFC-FC curves, the blocking temperature (T_B_) of both IONPs and the hybrid nanoparticles was determined to be 250 K. This T_B_ for IONPs aligns with the findings of de la Presa et al. by considering the size of the nanoparticles [42]. No significant change in T_B_ was observed in the hybrid phase, confirming that the magnetic properties of the IONPs remain largely unaffected by the presence of GNRs. This could be attributed to weak interactions between the two components, possibly due to the type of ligand used, the interparticle distance, or other factors. It is important to note that the weak magnetic coupling between Au and γ-Fe_2_O_3_ leaves the blocking temperature essentially unchanged.

### 4.2. Heating Efficiency of Nanoparticles

In hyperthermia, the thermal efficiency of nanoparticles is expressed by the Specific Absorption Rate (SAR). SAR is a measure of the rate at which energy is absorbed per unit time by a material when exposed to an electromagnetic field or laser irradiation. This heat generation is essential for hyperthermia treatment in cancer therapy, where elevated temperatures help to destroy cancer cells selectively. SAR is fundamental in determining the dosage and effectiveness of hyperthermia treatments. High SAR values indicate efficient heating, potentially allowing lower doses of nanoparticles and lower AMF, thus reducing the likelihood of unwanted heating in surrounding tissues. This selectivity is crucial for maximizing therapeutic benefits and minimizing side effects.

The energy dissipation in MHT primarily occurs through two relaxation mechanisms: Neel and Brownian relaxation. In Neel relaxation, the magnetic moment of the nanoparticle rotates within the particle’s crystal lattice. In Brownian relaxation, the entire nanoparticle rotates within the surrounding medium.

In MHT, the specific absorption rate (SAR) is influenced by a combination of factors related to the magnetic field, the properties of the nanoparticles, and the surrounding medium. Magnetic field parameters such as amplitude (H) and frequency (f) play a critical role: higher amplitudes and frequencies generally increase SAR, but must remain within clinically safe limits to avoid damaging healthy tissues; typically, frequencies should be kept below 1 MHz [11]. The properties of the nanoparticles themselves are equally important; materials like iron oxide (Fe_3_O_4_ or γ-Fe_2_O_3_) are commonly used due to their efficient heat generation [55], and optimal heating is often observed in particles with sizes between 5 and 30 nm, where superparamagnetic behavior at room temperature dominates. Particle shape also affects SAR, with anisotropic forms such as rods or cubes outperforming spherical particles in some cases [56]. Additionally, higher magnetic anisotropy in the particles enhances energy dissipation by resisting magnetic reorientation, thus increasing heat production. The medium in which the nanoparticles are dispersed also affects SAR; biological environments with blood flow or tissue can dissipate heat more quickly, reducing local temperature rise. Lastly, while increasing nanoparticle concentration tends to enhance SAR, excessive concentration may cause particle aggregation and lower heating efficiency due to dipolar interactions among closely spaced particles [42].

In the case of photothermia, the SAR also plays a relevant role, and the heating efficiency depends on several intrinsic and extrinsic factors, such as particle composition, size, concentration, laser intensity, etc. [44].

#### SAR Calculation

The SAR can be experimentally determined by monitoring the temperature increase in the nanoparticle suspension over time, given by

$$\mathrm{SAR}=C\;\frac{dT}{dt}$$

$$C=\frac{C_{w}\rho_{w}}{n_{p}(\frac{mg}{ml})}$$

where *C_w_* is heat capacity of water (4.184 J/gK); *ρ_w_* is density of water; *n_p_* is the total nanoparticles mass concentration in the suspension; and $\frac{dT}{dt}$ is the initial rate of temperature increase (K/s) slope of the temperature rise per second. The temperature rate should be monitored in an adiabatic environment in order to get a trustworthy SAR. However, as the measurement is not under adiabatic conditions, there are several fitting methods that can be used to correct for heat exchange with the surroundings [57,58].

### 4.3. Magnetic Hyperthermia

As explained in the synthesis section, magnetic nanoparticles were first synthesized separately and in subsequent steps were attached to GNRs with the help of ligands. The goal and novelty of this research is to combine magnetic hyperthermia and optical photothermal in order to increase the heat generation power of nanostructures. To optimize the SAR of magnetic nanoparticles, it is essential to first identify the field strength and frequency at which these nanoparticles exhibit their maximum SAR. This ensures efficient energy conversion and effective hyperthermia performance. Because two magnetic hyperthermia devices were available in the laboratory, it was possible to test a different range of alternating magnetic fields with different frequencies for IONPs.

**Figure 8:**
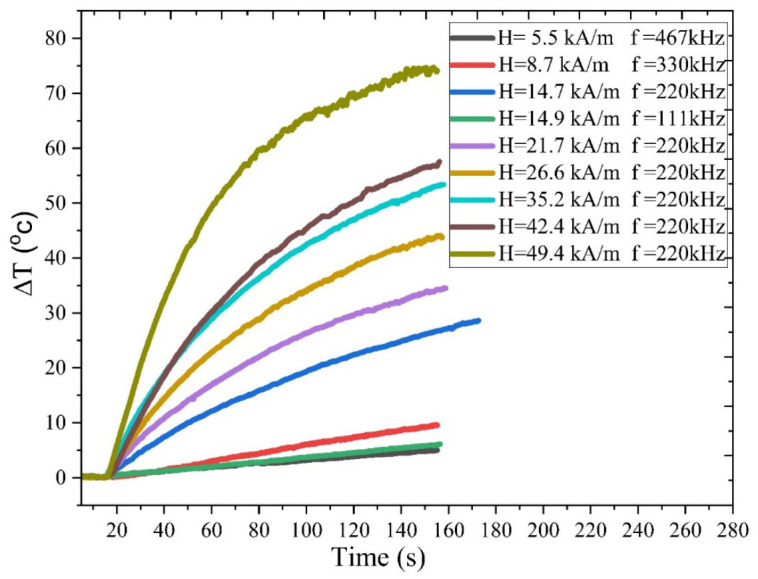
Temperature increases in the γ-Fe_2_O_3_ nanoparticles under different magnetic fields and frequencies within 150 s.

As can be seen in Figure 8, IONPs with a concentration of 1.5 mg/mL have been subjected to different alternating magnetic amplitudes and frequencies, and their best performance was obtained at a field of 49.4 kA/m and a frequency of 220 kHz. Considering the discomfort factor, this AMF is not allowed for biological applications [23]. For this purpose, Table 1 lists the permissible values of the magnetic field and frequency in living tissue according to the discomfort factor [11].

By examining Figure 8 and Table 1, it becomes evident that the magnetic field conditions yielding the highest temperature increases—field amplitudes of 26.6, 35.2, 42.4, and 49.4 kA/m at 220 kHz—are not suitable for in vivo applications, since in all these cases the H × f product exceeds the Atkinson–Brezovich criterion (H × f ≤ 5 × 10^9^ A·m^−1^·s^−1^). At the working frequency of 220 kHz, this criterion restricts the field amplitude to approximately 22.7 kA/m, so that the highest clinically compatible condition explored here is 21.7 kA/m (H × f = 4.8 × 10^9^ A·m^−1^·s^−1^). These elevated field strengths exceed the safety limits generally accepted for clinical use, as they may induce discomfort, unwanted tissue heating, or even damage to healthy tissue. For biological applications, especially in human medicine, magnetic field amplitudes are typically limited to around 31.8 kA/m at 100 kHz or less to ensure patient safety and regulatory compliance.

In this context, the selection of field parameters must balance heating efficiency with biocompatibility. According to Linear Response Theory [59], SAR is a key indicator of heating efficiency that scales quadratically with the amplitude of the magnetic field and linearly with its frequency. This means that small increases in field amplitude can lead to disproportionately large increases in SAR, while increasing frequency provides a linear improvement in heating efficiency.

This relationship is particularly evident when comparing experiments conducted at nearly identical field amplitudes (14.7 kA/m and 14.9 kA/m), but at substantially different frequencies (220 kHz and 110 kHz, respectively). Despite similar field strengths, the measurement taken at 220 kHz shows significantly higher heating efficiency, underscoring the critical role of frequency in optimizing magnetic hyperthermia. These findings highlight the importance of carefully selecting magnetic field parameters to maximize therapeutic outcomes while ensuring patient safety, especially when transitioning from in vitro experiments to clinical or in vivo applications.

The optimal heat generation capacity of the synthesized IONPs has now been determined, identifying the specific field strengths and frequencies at which they are most effective for combined photothermal and magnetic hyperthermia applications.

Up to this point, we have exclusively focused on the magnetic hyperthermia properties of the nanoparticles within the hybrid system, measuring their heating behavior under an alternating magnetic field and frequency optimized for biological applications. In the following sections, we will delve into the optical aspects of the research, examining factors that influence the optical properties of the hybrid nanostructure.

### 4.4. Photothermia

The efficiency of plasmonic nanoparticles in heat generation is governed by a complex interplay of factors, starting with the intrinsic properties of the nanoparticles themselves. The choice of material plays a central role, as metals such as gold, silver, and copper exhibit distinct plasmonic resonances and light absorption characteristics that directly determine their photothermal response. In addition, the size and shape of the nanoparticles are critical parameters. Different morphologies—including spheres, rods, and star-shaped structures—strongly influence light scattering and absorption processes. By carefully tuning these geometrical features, the photothermal performance of plasmonic nanoparticles can be significantly enhanced [60,61,62,63].

Equally important are the characteristics of the laser source used to excite the nanoparticles. The laser wavelength should ideally be matched to the plasmonic resonance of the nanoparticles in order to maximize light absorption and heat generation. Laser intensity is another key parameter: although higher intensities can enhance heat production, they must be carefully regulated to prevent damage to either the nanoparticles or the surrounding biological or synthetic medium. In addition, the pulse duration plays a crucial role in thermal behavior. Short laser pulses tend to induce highly localized heating, whereas longer pulses promote broader heat diffusion within the medium [64,65,66].

Environmental conditions further affect the heat generation process. The thermal properties of the medium surrounding the nanoparticles, such as their conductivity and heat capacity, govern how quickly heat is dissipated. Additionally, elevated ambient temperatures can alter the optical properties of plasmonic nanoparticles, potentially reducing their efficiency by shifting or broadening their resonance peaks [67,68].

Nanoparticle concentration also plays a crucial role. A higher density of nanoparticles in the solution typically leads to greater overall heat generation. However, excessively high concentrations may hinder light penetration and promote aggregation, both of which can diminish photothermal efficiency. Finding the right balance is essential for optimal performance [69].

Finally, interparticle interactions must be considered. When nanoparticles are closely spaced, their electromagnetic fields can couple, enhancing both light absorption and heat production. However, excessive coupling may lead to plasmon quenching, reducing the overall heating effect. Therefore, controlling the spacing and arrangement of nanoparticles is vital for achieving efficient and predictable thermal responses.

**Figure 9:**
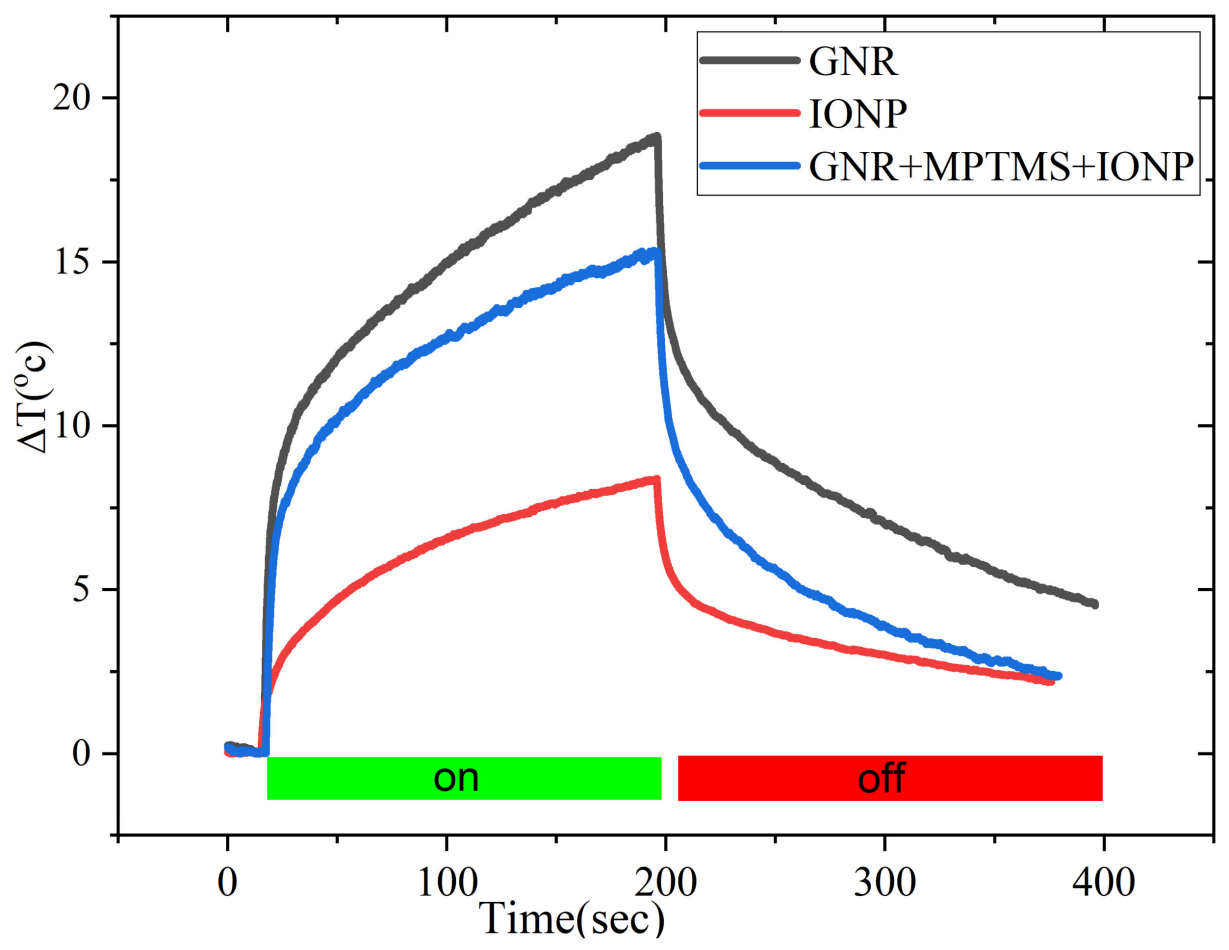
Temperature increases profiles (in °C) of GNRs, IONPs (γ-Fe_2_O_3_), and GNRs-iron oxide hybrid nanoparticles (GNR + MPTMS + IONP) just under 808 nm laser irradiation (optical excitation only).

To investigate the photothermal capabilities of hybrid nanoparticles, an appropriate light wavelength must be selected, and since GNRs act as the light absorber in the hybrid nanostructure, the wavelength must be selected close to the maximum absorption of the GNRs. The maximum absorption of GNRs according to the UV spectrum in Figure 1 is at 827 nm. Therefore, a laser with a wavelength of 808 nm could be the best choice for the photothermal process. The safe irradiance range for an 808 nm laser in photothermal therapy on living tissue, particularly in nanomedicine in vivo studies, is typically 0.3 to 1.5 W/cm^2^ [70,71,72]. Therefore, a laser with a wavelength of 808 nm and a power of 200 mW and 0.8 W/cm^2^ laser intensity was selected for photothermal tests. To isolate the photothermal contribution, each sample (1.5 mg/mL GNRs, 1.5 mg/mL γ-Fe_2_O_3_, and 3 mg/mL GNR + MPTMS + IONP) was individually exposed to 808 nm laser irradiation. The resulting temperature profiles are depicted in Figure 9.

All samples exhibited a temperature increase upon laser irradiation, confirming the conversion of light energy into heat. Notably, the sample containing only GNRs demonstrated the most significant temperature rise, attributed to the strong photothermal effect of GNRs, which efficiently absorb and convert incident laser light into heat. The IONPs (γ-Fe_2_O_3_) also exhibited a temperature increase, although to a lesser extent, indicating a lower light-to-heat conversion efficiency compared to GNRs [26]. The hybrid sample (GNR + MPTMS + IONP) showed an intermediate temperature rise, suggesting that the covalent attachment of IONPs to GNRs via the MPTMS ligand may have influenced the overall photothermal conversion efficiency of the system. These findings confirm the intrinsic photothermal properties of GNRs and underscore the potential to integrate GNRs with IONPs to develop hybrid systems with enhanced heat-generation capabilities. Figure 1 shows that the physical mixture of GNRs and γ-Fe_2_O_3_ preserves the longitudinal plasmon peak of the pure GNRs; the covalently linked hybrid shows a red-shifted, broadened, and less intense peak. This confirms that the covalent MPTMS linkage—rather than the mere presence of iron-oxide particles—modifies the local dielectric environment of the GNRs, lowering their absorbance at 808 nm. At an identical gold-nanorod concentration, this reduced absorbance accounts for the lower laser-only heating of the hybrid observed in Figure 9.

### 4.5. Combined Magnetic Hyperthermia and Photothermia

To investigate the heat generation capabilities of hybrid nanoparticles, three samples were prepared: (1) γ-Fe_2_O_3_ with concentration of 1.5 mg/mL, (2) GNRs chemically linked to IONPs via (3-mercaptopropyl)trimethoxysilane (MPTMS) ligands (GNR + MPTMS + IONP) with concentration of 3 mg/mL (1.5 mg/mL γ-Fe_2_O_3_ + 1.5 mg/mL GNRs), and (3) a physical mixture of GNRs and IONPs without chemical bonding (GNR + IONP) with total concentration of 3 mg/mL (1.5 mg/mL γ-Fe_2_O_3_ + 1.5 mg/mL GNRs). Each sample was simultaneously subjected to near-infrared (NIR) laser irradiation (808 nm, 200 mW) and a high-frequency alternating magnetic field. The temperature evolution was monitored using a thermal imaging camera (resolution 0.1 °C).

The heating protocol consisted of a 180 s “on” period during which both stimuli were applied, followed by a 180 s “off” period to allow passive cooling. The SAR was determined using the exponential decay fitting method, as described in previous studies [73,74,75].

**Figure 10:**
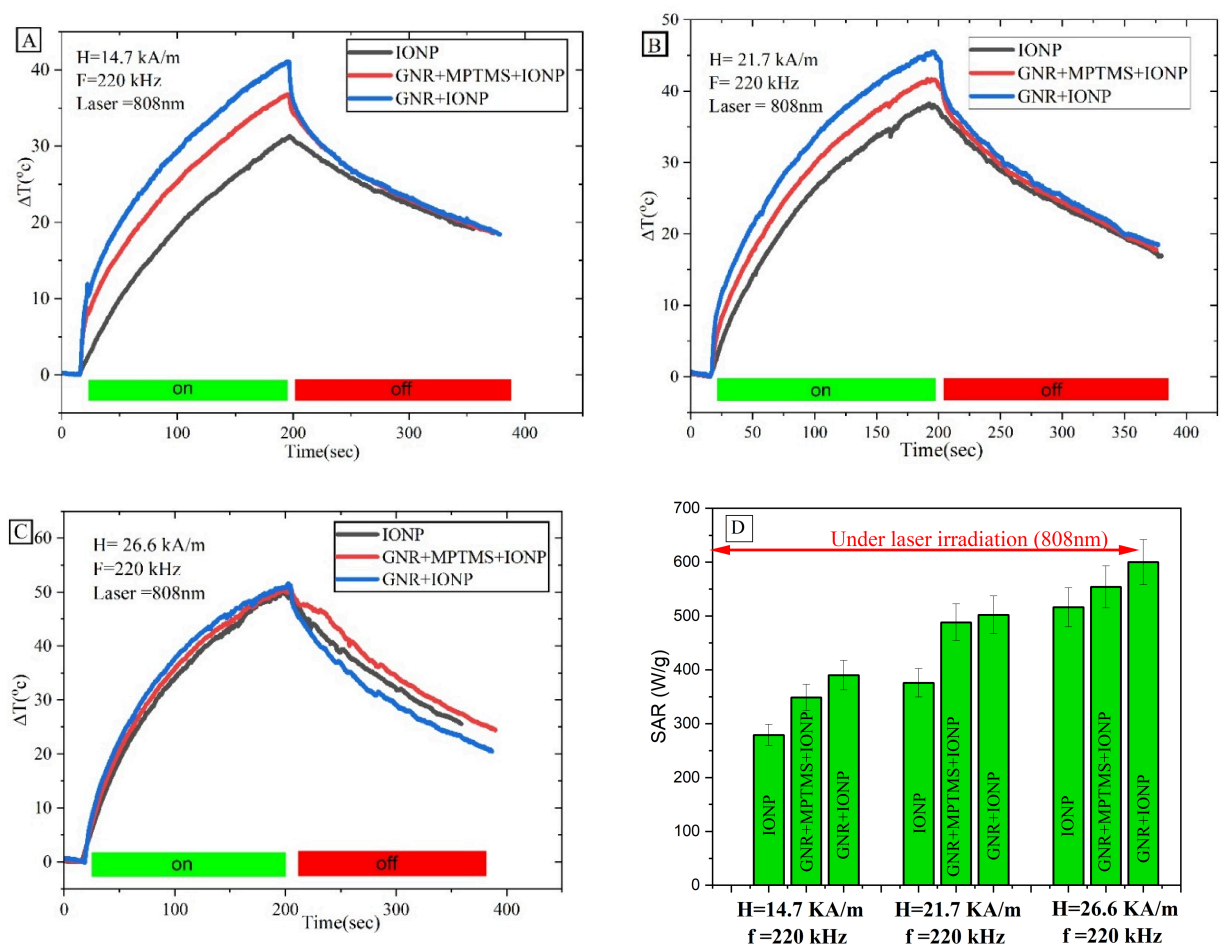
Temperature increase under simultaneous magnetic hyperthermia and photothermal of IONPs, GNR + MPTMS + IONP, and γ-Fe_2_O_3_ nanoparticles mixed with GNRs (GNR + IONP), these samples were exposed to 808 nm laser radiation and magnetic field of 14.7 kA/m (**A**), 21.7 kA/m (**B**), and 26.6 kA/m (**C**). (**D**) SAR column chart for simultaneous magnetic hyperthermia and photothermia. All SAR values are normalized to the total nanoparticle mass concentration of each suspension (1.5 mg·mL^−1^ for γ-Fe_2_O_3_; 3 mg·mL^−1^ for GNR + MPTMS + IONP and GNR + IONP). Error bars represent the propagation of the nanoparticle mass-concentration uncertainty into the SAR.

The GNR + MPTMS + IONP sample represents a hybrid structure in which IONPs are covalently bonded to GNRs via the MPTMS linker, promoting efficient thermal coupling. In contrast, the GNR + IONP sample involves only physical mixing, without chemical interaction between components. For comparative analysis, γ-Fe_2_O_3_ nanoparticles alone were also evaluated under identical conditions. The thermal performance of all three formulations is presented in Figure 10, highlighting the impact of nanoparticle architecture on photothermal and magnetothermal characterization.

The analysis of the temperature rise profiles depicted in Figure 10A–C reveals that the incorporation of GNRs significantly enhances the heat-generation efficiency of the nanoparticle systems. This improvement is attributed to the strong plasmonic absorption of GNRs under near-infrared (NIR) irradiation, which contributes to efficient photothermal conversion. However, a direct comparison between the (GNR + MPTMS + IONP) and (GNR + IONP) samples shows that their SAR values coincide within experimental error at the three field amplitudes tested (Figure 10D). The covalently bonded hybrid displays a nominally lower mean SAR, consistent with a partial restriction of the Brownian relaxation mechanism—a dominant contributor to magnetic heat dissipation—by the MPTMS linkage, but this difference lies within the experimental uncertainty. In addition, as previously described, the GNR + MPTMS+ IONP system is less efficient under laser irradiation than GNR + IONP, because the covalent binding of γ-Fe_2_O_3_ to GNRs through MPTMS affects the optical absorbance of the GNRs, as shown in Figure 1.

In contrast, the physically mixed sample, in which GNRs and γ-Fe_2_O_3_ nanoparticles are not chemically linked, allows greater freedom of motion for the magnetic nanoparticles and preserves the higher optical absorbance of the GNRs (see Figure 1). These observations are consistent with previous reports by Ovejero et al. [28], who described reduced heat generation in silica-coated magnetic nanoparticles chemically linked to GNRs, presumably due to a deterioration of the optical properties of the GNRs.

Additionally, Figure 10D illustrates the dependence of the specific absorption rate (SAR) on the applied magnetic field strength. As expected, SAR values increase with increasing field amplitude, in agreement with theoretical predictions indicating that stronger magnetic fields induce higher magnetic losses and, consequently, greater thermal energy dissipation in magnetic nanoparticle systems.

Collectively, these results demonstrate that the combination of magnetic and photothermal stimuli can effectively enhance the heating performance of hybrid nanomaterials. While the presence of GNRs markedly boosts overall thermal output, the nature of the interparticle linkage does not produce a measurable difference in the final SAR values. The physical mixture displays a nominally higher mean SAR, but the difference lies within experimental error. It should nevertheless be emphasized that the SAR measurements, now including experimental error bars (Figure 10D), show that the covalently linked (GNR + MPTMS + IONP) and physically mixed (GNR + IONP) samples reach equal SAR values within experimental uncertainty under simultaneous excitation. The two configurations are therefore equivalent in heating performance within the resolution of our measurements. This equivalence must be weighed against a decisive functional advantage of the covalent hybrid: in a physical mixture, the GNRs and the magnetic nanoparticles remain independent, whereas many envisaged applications require the two components to behave as a single entity. In particular, magnetic guidance and magnetically driven accumulation of the nanostructure at the target site—as well as the co-localization of the plasmonic and magnetic heating sources within the same object—can only be achieved when the components are physically joined, as in the covalently linked hybrid. In a simple mixture, an applied magnetic field would displace the IONPs while leaving the GNRs behind, so that the plasmonic and magnetic functionalities would become spatially decoupled. The covalent MPTMS linkage therefore provides—without any measurable penalty in heating efficiency—the structural integrity required for magnetic manipulation, targeting, and reliable dual-mode operation of the whole nanostructure.

To place these results in a broader context, Table 2 compares the SAR of our hybrid nanostructure with values reported for other GNR- and iron-oxide-based systems operated under the same dual (magnetic field plus NIR laser) excitation. To make the comparison meaningful, only studies using a laser within the first biological window and intensities comparable to ours were retained, and the magnetic-field conditions of each work were assessed against the Atkinson–Brezovich criterion (H × f ≤ 5 × 10^9^ A·m^−1^·s^−1^) to judge their compatibility with clinical use. Two groups emerge from this analysis. The first comprises the studies that respect the biological limit [25,27]: although clinically applicable in principle, their SAR values (~300 and ~50 W·g^−1^, respectively) remain well below the ~500 W·g^−1^ reached by our hybrid under comparably safe conditions (22 kA/m, 220 kHz; H × f = 4.8 × 10^9^ A·m^−1^·s^−1^). The second group gathers the studies reporting SAR values similar to or higher than ours [13,26,28]; however, all of them operate outside the safety window, either because the H × f product exceeds the Atkinson–Brezovich limit [13,26] or because the field amplitude (52 kA/m in Ref. [28]) far surpasses the amplitude tolerable in vivo. Notably, the only system that exceeds ours in absolute terms (Ref. [28], ~700 W·g^−1^) does so under a field that could not be applied in an actual cancer treatment. Overall, our hybrid nanostructure offers the most favorable balance between heating power and clinical feasibility: among the systems that stay within the biologically permissible field conditions, it delivers the highest SAR, whereas the few systems with comparable or superior SAR are ruled out from clinical translation by their unsafe operating conditions.

## 5. Conclusions

This study reports the synthesis and structural, optical, and magnetic characterization of a magneto-plasmonic hybrid nanostructure in which GNRs are linked to γ-Fe_2_O_3_ nanoparticles through (3-mercaptopropyl)trimethoxysilane (MPTMS), together with its heating response under magnetic, optical, and simultaneous dual-mode excitation.

Several independent observations support the formation of the hybrid: the longitudinal plasmon resonance of the GNRs red-shifts from 751 to 827 nm upon conjugation, a shift absent in the linker-free mixture; the XRD pattern of the linked sample displays the γ-Fe_2_O_3_ and Au reflections together with additional low-angle features; and TEM shows the γ-Fe_2_O_3_ nanoparticles decorating the rods. Magnetometry confirms superparamagnetic behavior at room temperature and a blocking temperature (250 K) essentially unchanged with respect to the bare γ-Fe_2_O_3_, indicating that the linkage to the gold does not alter the intrinsic magnetic response of the iron oxide.

Under simultaneous 808 nm irradiation and an alternating magnetic field, the hybrid reaches a SAR of 488 ± 34 W·g^−1^ at 21.7 kA/m and 220 kHz, well above the temperature rise obtained under laser irradiation alone and under field conditions that satisfy the Atkinson–Brezovich criterion (H × f = 4.8 × 10^9^ A·m^−1^·s^−1^). As shown in Table 2, this is the highest SAR among the compared systems operating within the clinically permissible field window.

The covalently linked hybrid and the physical mixture reach equal SAR within experimental error (Figure 10D): the modification of the GNR absorbance and the partial restriction of Brownian relaxation introduced by the linkage are too small to produce a measurable difference. The covalent architecture, therefore, costs no heating power while providing what a mixture cannot—a single mechanically joined entity, a prerequisite for magnetic guidance and for keeping the plasmonic and magnetic sources co-localized at the target site.

These results constitute a physicochemical proof of concept established in aqueous suspension. The proposed GNR + MPTMS + IONP hybrid nanostructure represents a promising candidate for rapid, efficient, and controllable hyperthermia treatment; its dual-response capability offers a pathway toward more localized and effective cancer therapies. Cytotoxicity, colloidal stability, and protein-corona formation in biological media, haemocompatibility, cellular uptake, and in vitro/in vivo validation remain necessary steps before any therapeutic use can be claimed.

## Figures and Tables

**Table 1:** Permissible alternating magnetic field values and frequencies for use in living tissue according to the Atkinson–Brozovic limit. 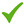 suitable, 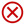 non-suitable.

H (kA/m)	f (kHz)	SAR (W/g)	H × f ≤ 5 × 10^9^ (A/ms)	Safe for Clinical Treatments
5.5	467	25	2.6 × 10^9^	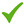
8.7	330	73	2.9 × 10^9^	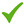
14.7	220	209	3.2 × 10^9^	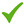
14.9	111	31	1.7 × 10^9^	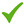
21.7	220	408	4.8 × 10^9^	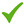
26.6	220	418	5.9 × 10^9^	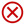
35.2	220	480	7.8 × 10^9^	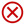
42.4	220	502	9.4 × 10^9^	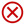
49.4	220	583	11 × 10^9^	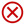

**Table 2:** Comparison of SAR values reported in the literature for magneto-plasmonic systems operated under simultaneous magnetic-field and laser excitation, together with the value obtained in this work. Only studies using an NIR laser in the first biological window and laser intensities comparable to ours were included. For each entry, the applied magnetic-field conditions were checked against the Atkinson–Brezovich limit (H × f ≤ 5 × 10^9^ A·m^−1^·s^−1^): a green check mark denotes conditions compatible with clinical use, and a red cross denotes conditions that exceed the biologically safe range. SAR values were estimated from the corresponding figures of each reference and should therefore be taken as approximate.

References	H (kA/m)	f (kHz)	Laser Intensity (W/cm^2^)	SAR (W/g)	Safe for Clinical Treatment
This work	22	220	0.8	488 ± 34	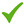
Ref. [25]	12	180	0.3	~300	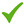
Ref. [26]	14	470	0.5	~400	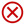
Ref. [27]	17	100	0.3	~50	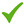
Ref. [13]	20	900	0.3	~200	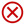
Ref. [28]	52	50	0.3	~700	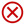

## Data Availability

The raw data supporting the conclusions of this article will be made available by the authors on request.

## Supplementary Materials

The following supporting information can be downloaded at: https://www.mdpi.com/article/10.3390/nano16150938/s1. Additional TEM micrographs of the bare GNRs (Figure S1), the IONPs (Figure S2), the GNR + MPTMS + IONP hybrid after the complete washing protocol (Figure S3), and the GNR + IONP physical mixture (Figure S4).
