# Supplementary material for: Hybrid Magneto-Plasmonic Nanostructures for Enhanced Dual-Mode Hyperthermia
Source: Nanomaterials (Basel). 2026 Jul 29;16(15):938. doi: 10.3390/nano16150938 (PMC13468272; doi:10.3390/nano16150938)
Supplement: Supplementary file 1 [file nanomaterials-16-00938-s001.zip › nanomaterials-4409308-supplementary.pdf]

SUPPORTING INFORMATION: Hybrid Magneto-Plasmonic Nanostructures for Enhanced Dual-Mode Hyperthermia

Amirhossein Sanchooli[a] and Patricia de la Presa[b,c]

[a] Department of Physics, Faculty of Science, University of Kurdistan, Sanandaj 66177-15175, Iran.

[b] Institute of Applied Magnetism, UCM, A6 22.500 km, 28230 Las Rozas, Spain

[c] Department of Material Physics, Complutense University of Madrid, Madrid 28040, Spain.

Email: pmpresa@ucm.es

TEM Images

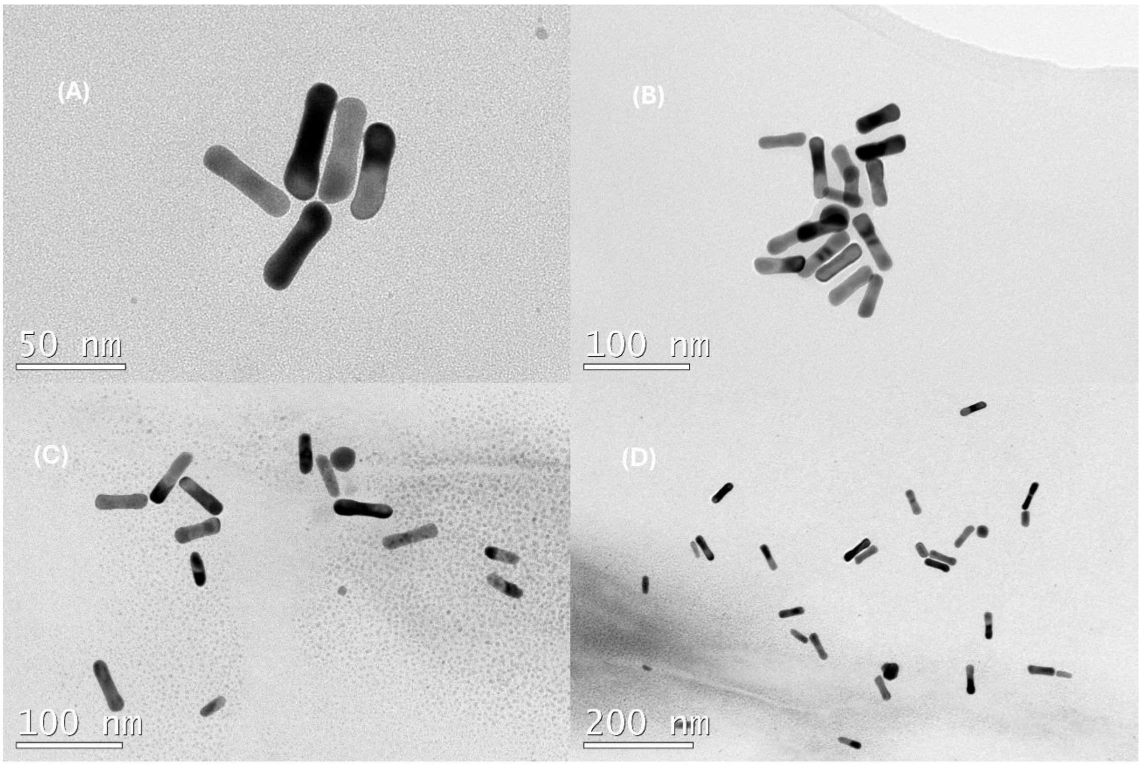

Figure S1: Representative TEM micrographs of the bare GNRs at different magnifications; scale bars are (A) 50 nm, (B) and (C) 100 nm, (D) 200 nm.

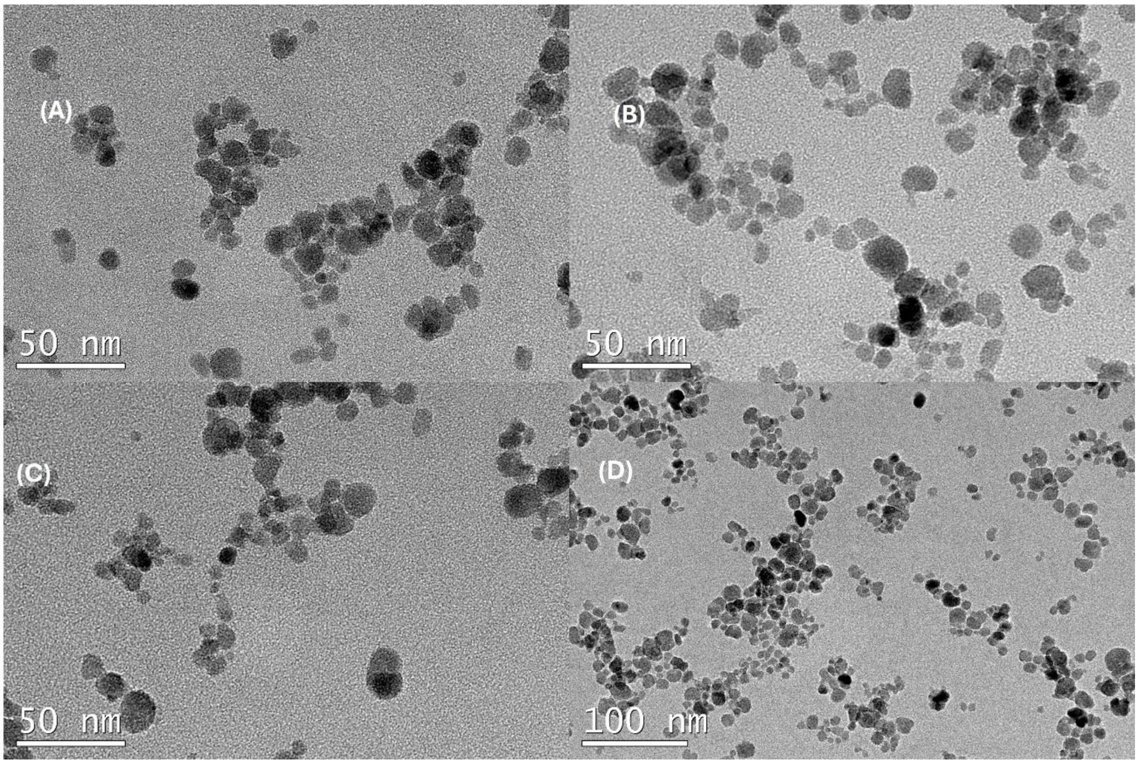

Figure S2: Representative TEM micrographs of the bare IONPs at different magnifications, the scale bars are (A), (B) and (C) 50 nm and (D) 100 nm

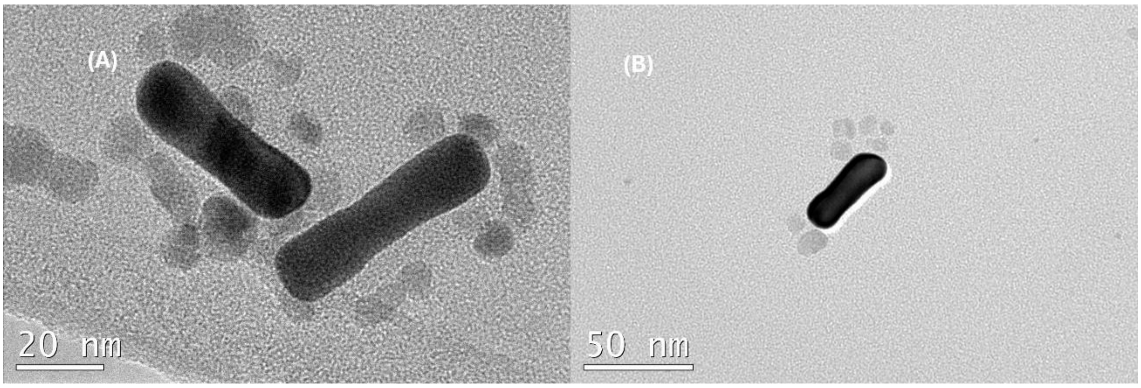

Figure S3: Representative TEM micrographs of the bare GNR-MPMTS-IONP at different magnifications, the scale bars are (A) 20 nm and (B) 50 nm.

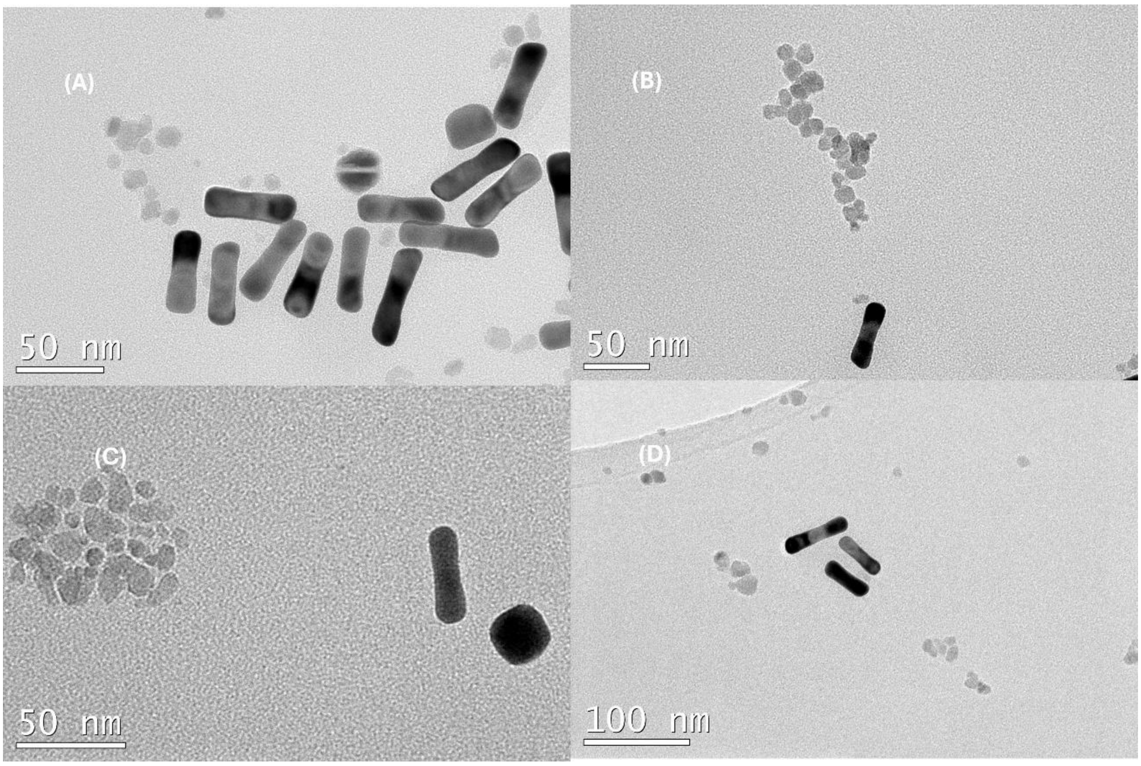

Figure S4: Representative TEM micrographs of the bare GNR+IONP at different magnifications, the scale bars are (A), (B) and (C) 50 nm and (D) 100 nm
